# Comparative cost-effectiveness of cross-sectional imaging strategies in the diagnosis of intervertebral disc extrusion in dogs: a United Kingdom-based decision-analytic study

**DOI:** 10.1093/jvimsj/aalag016

**Published:** 2026-02-23

**Authors:** Daniel Low

**Affiliations:** Small Animal Surgery, Frank. Pet Surgeons, Leeds, United Kingdom; Small Animal Surgery, Swift Referrals, Wetherby, United Kingdom

**Keywords:** cost-effectiveness, intervertebral disc extrusion, neuroimaging, neurology

## Abstract

**Background:**

Cross-sectional imaging, with particular regard to computed tomography (CT) and magnetic resonance imaging (MRI), has been shown to be accurate in the diagnosis of intervertebral disc extrusion (IVDE) in dogs, with MRI having higher diagnostic accuracy. However, the cost-effectiveness of veterinary diagnostic imaging has not been investigated.

**Hypothesis/Objectives:**

Comparative evaluation of the cost-effectiveness of 5 cross-sectional imaging strategies in the diagnosis of thoracolumbar IVDE.

**Animals:**

No live animals were used.

**Methods:**

A probabilistic decision-analytical model was developed based on a hypothetical cohort of dogs suspected to have thoracolumbar IVDE. Five imaging strategies based on a combination of noncontrast CT, CT-myelography (CTM), and MRI were tested (CT-only, noncontrast CT only; conditional-CTM, noncontrast CT followed by CT-myelography if nondiagnostic; unconditional-CTM, noncontrast CT followed by CT-myelography; conditional-MRI, noncontrast CT followed by MRI if nondiagnostic; MRI-only, MRI only). Effectiveness was defined as the probability of a correct diagnosis.

**Results:**

Across probabilistic simulations, CT-only and unconditional-CTM were consistently less effective and more costly than other options and therefore were never preferred (strictly dominated). Magnetic resonance imaging-only was less cost-effective than strategies based on noncontrast CT (extendedly dominated). Conditional-CTM and conditional-MRI provided the best balance of diagnostic accuracy and cost (the non-dominated efficient set of strategies).

**Conclusions and clinical importance:**

Conditional imaging strategies beginning with noncontrast CT and escalating only if nondiagnostic were the most cost-effective strategies in diagnosing thoracolumbar IVDE in dogs. An MRI-only strategy was rarely cost-effective despite similar diagnostic sensitivity. From this decision-analytic modeling study, strategic use of cross-sectional imaging in the diagnosis of thoracolumbar IVDE has the potential to optimize the use of finite resources.

## Introduction

Thoracolumbar intervertebral disc extrusion (IVDE) is one of the leading causes of acute spinal cord injury and pelvic limb dysfunction in dogs,^1^ with an approximate annual incidence of 2.4% in predisposed breeds.^2^ First described using myelography over half a century ago,^3,4^ IVDE is now more commonly diagnosed using cross-sectional imaging modalities such as computed tomography (CT) and magnetic resonance imaging (MRI).^5,6^ Before planning any potential decompressive surgery, accurate identification of IVDE is important to guide additional treatment decisions.^5^

Computed tomography with or without subarachnoid contrast administration is important in the diagnostic evaluation of intervertebral disc disease in dogs, including IVDE.^5^ Noncontrast CT has been reported to have a sensitivity of approximately 81%, approaching 100% in the subgroup of chondrodystrophic dogs with mineralized intervertebral discs.^7,8^ With the addition of subarachnoid contrast medium, CT-myelography has improved sensitivity compared with noncontrast CT, with a reported sensitivity of approximately 97%.^9^ However, the use of myelography is associated with adverse events such as seizures, which may increase the morbidity of the procedure.^10–12^ Magnetic resonance imaging is considered the gold standard imaging modality to diagnose IVDE in dogs,^5^ with reported sensitivities over 98.5%.^13^ However, the longer acquisition time and extended duration of general anesthesia associated with MRI can contribute to higher overall costs compared with CT-based techniques.^14^ At present, noncontrast CT, CT-myelography, and MRI are all recognized as reasonable options for the diagnosis of thoracolumbar IVDE in dogs, with MRI having higher diagnostic accuracy.^5^

Although numerous studies have explored the diagnostic performance and clinical utility of CT, CT-myelography, and MRI, no studies have systematically compared their cost-effectiveness in diagnosing thoracolumbar IVDE in dogs. Over the past decade, medical inflation in both human and veterinary healthcare has consistently outpaced the consumer price index, placing increasing strain on both single-payer and privately funded healthcare systems.^15–18^ Many national health systems have dedicated agencies to evaluate the cost-effectiveness of diagnostic and therapeutic interventions, with the goal of optimizing resource allocation and ensuring equitable access to high-quality healthcare.^19,20^ Even when a diagnostic test is clinically effective, it cannot be assumed to be cost-effective in all contexts.^21^ Quantitative cost-effectiveness analysis scrutinizes interventions to identify approaches that are not only clinically effective but also associated with efficient healthcare service provision.^22^ Diagnostic imaging plays a central role in medical decision-making, and clinicians should demand proof of value before widespread adoption of new diagnostic imaging modalities.^23^

Veterinary neurology services often require advanced imaging and specialist expertise, which may limit accessibility to the general population of animal owners. Although veterinary data are unavailable, advances in cross-sectional imaging technologies have been a leading driver of medical inflation in health care for humans.^24^ Because the veterinary diagnostic imaging market is forecast to reach US $3.15 billion by 2030,^25^ and in the face of economic pressures, resource limitations, and external regulatory scrutiny,^26–28^ comparative cost-effectiveness of cross-sectional imaging strategies in the diagnosis of IVDE becomes increasingly relevant to decision-making. A recent consensus statement identified the need to develop cost-effectiveness studies to support the diagnosis of IVDE.^14^ The study reported here aimed to use a decision-analytic model to compare the cost-effectiveness of 5 cross-sectional imaging strategies in the diagnosis of thoracolumbar IVDE in dogs.

## Materials and methods

### Model structure and imaging strategies

**Figure 1:**
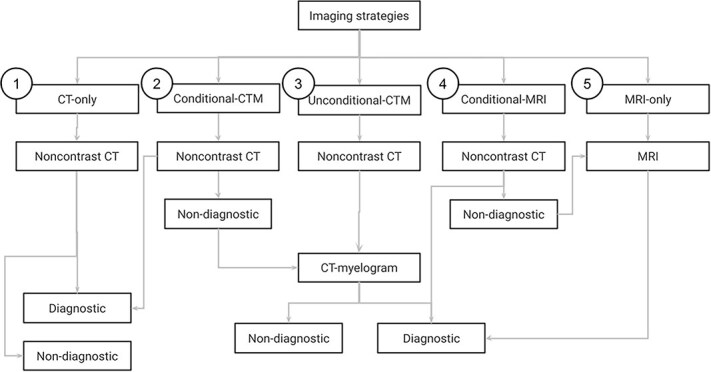
Decision-analytic model structure showing the decision branches of 5 cross-sectional imaging strategies in the diagnosis of thoracolumbar IVDE in dogs. Abbreviations: CT = computed tomography; CTM = computed tomography-myelogram; MRI = magnetic resonance imaging.

A decision-analytic model^23^ was developed to assess 5 cross-sectional imaging strategies in a hypothetical cohort of dogs with suspected thoracolumbar IVDE (Figure 1). A healthcare sector perspective was adopted.^29^ Five cross-sectional imaging strategies to diagnose thoracolumbar IVDE were defined, based on previously reported clinical approaches to cross-sectional imaging (Table 1). In the conditional-CTM strategy, noncontrast CT was used as the initial diagnostic test and conditionally followed up with CT-myelography if noncontrast CT findings were inconclusive.^30^ In the unconditional-CTM strategy, noncontrast CT was used as the initial diagnostic test and unconditionally followed up with CT-myelography.^31^ In the conditional-MRI strategy, noncontrast CT was used as the initial diagnostic test and conditionally followed up with MRI if noncontrast CT findings were inconclusive, according to the base sensitivity of noncontrast CT.^32^ Effectiveness was defined as the probability of a correct diagnosis. The study horizon was limited to the point of diagnosis, only including analysis of the costs of any cross-sectional imaging used and the costs of treating direct complications arising from myelography, if applicable. Given the short-term horizon, no discounting was undertaken.

**Table 1:** Definition of the 5 cross-sectional imaging strategies tested.

**Strategy code**	**First diagnostic test**	**Second diagnostic test**
**CT-only**	Noncontrast CT	None
**Conditional-CTM**	Noncontrast CT	Conditional follow-up with CT-myelography if inconclusive
**Unconditional-CTM**	Noncontrast CT	Unconditional follow-up with CT-myelography
**Conditional-MRI**	Noncontrast CT	Conditional follow-up with MRI if inconclusive
**MRI-only**	MRI	None

Abbreviations: CT = computed tomography; CTM = computed tomography-myelogram; MRI = magnetic resonance imaging.

### Model parameters

Model input parameters included the diagnostic sensitivities of each imaging modality, the cost of each imaging modality, the complication rate of myelography, and the cost of treating myelography-related complications. Diagnostic sensitivity of CT was defined using a point estimate of 81%, reflecting the minimum sensitivity of CT in a mixed population of chondrodystrophic and non-chondrodystrophic dogs suspected to have thoracolumbar IVDE.^7,8^ This sensitivity was held constant across all imaging strategies. Diagnostic sensitivity of CT-myelography was defined as 97%.^9^ The complication rate of myelography was defined as 14%, reflecting a mid-range complication rate from the literature.^12^ In the conditional-CTM strategy, the conditional probability of performing CT-myelography after noncontrast CT was defined as 27% based on published clinical practice data.^30^ This probability reflects the proportion of dogs undergoing additional imaging after noncontrast CT for any reason, and includes nondiagnostic noncontrast CT studies as well as selected cases with diagnostic CT findings in which further localization or characterization of extradural compression was pursued. The cost of each diagnostic imaging procedure was estimated from a web search of publicly available procedural prices in the United Kingdom (UK), where available, and industry knowledge, where procedural prices were unavailable. All costs were calculated in British pounds. The costs of CT, CT-myelography, MRI, and treating myelography-related complications were estimated to be £1500, £300 in addition to CT, £2700, and £1000, respectively. For the conditional-CTM and conditional-MRI strategies, weighted averages were used to estimate costs, based on the probability of requiring a second imaging modality. Probabilistic sensitivity analysis (PSA) was performed using a 1000-iteration Monte Carlo simulation to account for input parameter uncertainty and to evaluate robustness of cost-effectiveness outcomes over a range of plausible input values. Stability of the main outcomes, cumulative costs and cumulative effectiveness, was visually assessed using convergence plots. All model inputs except for the diagnostic sensitivity of MRI were treated as stochastic variables. Cost parameters were sampled from a γ distribution with a coefficient of variation of 15.0%, reflecting moderate variation in cost estimates. Cost estimates were intentionally specified with wide uncertainty ranges to capture variation in clinical practice, including differences related to the use of sedation versus general anesthesia, particularly for CT-based imaging. The costs for sedation or general anesthesia were built-in and accounted for within the cost estimates and not as separate items. For each cost parameter, the shape (α) was computed as the reciprocal of the squared coefficient of variation, and the scale (θ) was computed as the product of the point estimate and the squared coefficient of variation. Probability parameters were sampled from a β distribution with a standard error of 0.05, which was used to compute the shape (α) and scale (θ). Magnetic resonance imaging was defined as the gold standard and had its diagnostic sensitivity held constant at 100.0%. Results of parameter sampling were reported as medians, quartiles, minima, and maxima.

### Cost-effectiveness analysis

In each simulation iteration of the PSA, costs and effectiveness of each strategy were recalculated based on sampled parameter values. For each strategy, distributions of expected cost and effectiveness were generated. Results were visualized using a cost-effectiveness plane, and comparative distributions were evaluated to determine dominance and relative value. CT-only was used as the reference strategy on the cost-effectiveness plane for having the lowest cost and sensitivity, allowing incremental cost-effectiveness assessment of all other strategies. Across the 1000 probabilistic simulations, dominance analysis was performed in each simulation to identify the economically dominant strategy. Strict dominance was defined as strategies that were more costly and less effective than another strategy. Strategies that were strictly dominated were excluded. Extended dominance was defined as strategies that were inefficient relative to a theoretical combination of 2 more efficient (non-dominated) strategies. Extendedly dominated strategies were excluded. Non-dominated strategies were defined as the remaining strategies that were neither strictly nor extendedly dominated, and formed the efficient set of strategies. Incremental cost-effectiveness ratios (ICERs) of non-dominated strategies were calculated by comparing each strategy to the next least costly alternative. The reference comparator to compute ICERs varied among simulations, depending on the results and dominance rankings between iterations.

The net monetary benefit (NMB) was calculated for each non-dominated strategy at cost-effectiveness thresholds (CET) ranging from £0 to £100 000 per additional correct diagnosis. Cost-effectiveness thresholds represent the amount a decision-maker is willing to pay for an additional correct diagnosis. Cost-effectiveness acceptability curves (CEACs) were generated by calculating, for each CET, the proportion of simulations in which each strategy had the highest NMB. Crossover points between CEACs were computed using linear interpolation to identify the CET where the most cost-effective strategy changed. The expected value of perfect information (EVPI) was calculated to quantify the value of eliminating decision uncertainty. At each cost-effectiveness threshold, the EVPI was computed as the difference between the mean NMB of the optimal strategy under perfect information and the NMB of the strategy with the highest expected NMB under current uncertainty.

### Statistical analysis

Data normality was assessed using the Shapiro–Wilk test. Parametric data were reported as mean and SD. Nonparametric data were reported as median and IQR. Statistical analysis and data visualization were performed with NumPy 2.0.2, Pandas 2.2.2, SciPy 1.16.0, and Matplotlib 3.10.0 in Python version 3.11.13.

## Results

**Figure 2:**
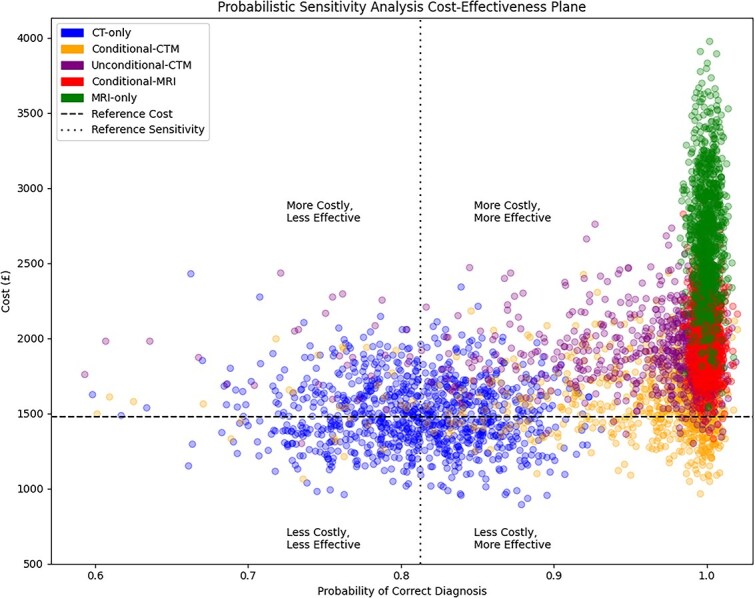
Cost-effectiveness plane showing the distribution of incremental costs and effects from PSA, comparing all imaging strategies relative to CT-only as the reference, with each point representing a single iteration. Abbreviations: CT = computed tomography; PSA = probabilistic sensitivity analysis.

The results of stochastic parameter sampling are shown in Table 2 and File S1, reflecting a range of plausible model input parameters. Results of the PSA were visualized on the cost-effectiveness plane, with CT-only as the reference strategy (Figure 2). All 4 non-reference strategies lay within the northeast and southeast quadrants.

**Table 2:** Results of stochastic parameter sampling.

**Parameter**	**Median**	**Range**	**Interquartile range**
**MRI sensitivity**	100.0%		
**Noncontrast CT sensitivity**	81.3%	59.8%-93.1%	77.9%-84.6%
**CT-myelogram sensitivity**	99.3%	60.9%-100.0%	96.6%-99.9%
**Myelography complication rate**	13.2%	3.2%-34.3%	10.2%-17.0%
**CT cost (£)**	1477.40	893.48-2430.54	1344.70-1645.56
**Myelography cost (£)**	298.82	148.42-449.62	265.97-330.34
**Cost of myelography complications (£)**	993.78	575.25-1531.75	889.75-1087.31
**MRI cost (£)**	2705.94	1543.21-3996.28	2453.34-2965.26

Abbreviations: CT = computed tomography; MRI = magnetic resonance imaging.

The convergence plots showed that cumulative costs and cumulative effectiveness stabilized by approximately 200-400 simulations, indicating that 1000 iterations were sufficient to provide robust results (File S2). Quantitative results of PSA are shown in Table 3. Computed tomography (CT)-only and unconditional-CTM were strictly dominated during PSA and were therefore not cost-effective under any probabilistic simulations. Magnetic resonance imaging (MRI)-only was extendedly dominated in the majority of PSA simulations, being placed on the efficiency frontier in only 31/1000 (3.1%) simulations. In contrast, conditional-CTM and conditional-MRI were non-dominated in 927/1000 (92.7%) and 969/1000 (96.9%) of simulations, respectively, comprising the efficient set under the tested parameter values. Conditional-CTM was associated with a median ICER of £695.25 (IQR, £563.75-£890.66) per additional correct diagnosis compared to CT-only. Conditional-MRI was associated with a median ICER of £55 501.35 (IQR, £11 684.53-£391 919.31) per additional correct diagnosis compared to conditional-CTM. Magnetic resonance imaging (MRI)-only was associated with a median ICER of £16 755.14 (IQR, £1643.34-£169 976.28) compared to conditional-CTM, although the low count of MRI-only being a non-dominated strategy limits the robustness of this ICER estimate.

**Table 3:** Incremental cost-effectiveness ratio of the extendedly dominated and non-dominated strategies, calculated relative to the next least costly non-dominated strategy in each simulation.

**Strategy code**	**Dominance result**	**ICER (£ per unit effectiveness)**	**Non-dominated count**
**Conditional-CTM**	Non-dominated	695.25 (563.75-890.66)	927 (92.7%)
**Conditional-MRI**	Non-dominated	55 501.35 (11 684.53-391 919.31)	969 (96.9%)
**MRI-only**	Extendedly dominated	16 755.14 (1643.34-169 976.28)	31 (3.1%)

Abbreviations: CTM = computed tomography-myelography; ICER = incremental cost-effectiveness ratio; MRI = magnetic resonance imaging.

**Figure 3:**
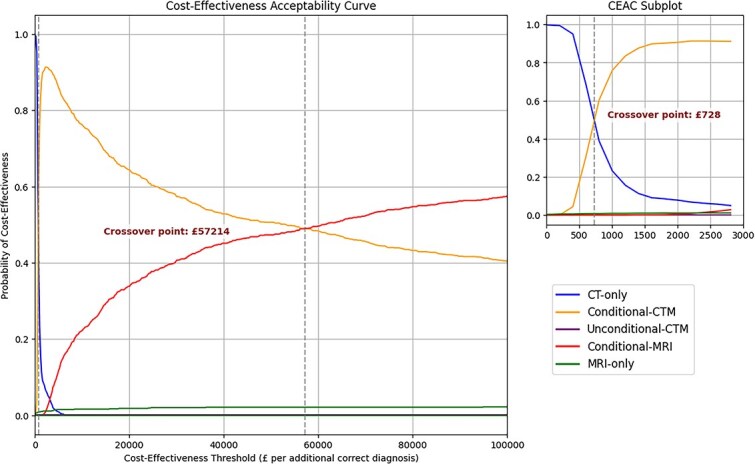
Cost-effectiveness acceptability curve showing the probability of each imaging strategy being cost-effective across a range of CET. Crossover points indicate thresholds where the economically preferred strategy changes, reflecting the impact of CET on optimal decision-making. Abbreviations: CET = cost-effectiveness thresholds.

The probability of each imaging strategy being cost-effective across a range of CETs is illustrated in the CEAC (Figure 3). At low CETs of < £728, where only minimal spending is justified for improved diagnostic accuracy, CT-only emerged as the strategy with the highest probability of being cost-effective despite being strictly dominated in PSA. Conditional-CTM was most likely to be cost-effective across intermediate CETs of £728-£57 214, where balanced diagnostic accuracy and cost was favored. At high CETs of > £57 214 where diagnostic accuracy was valued over costs, conditional-MRI was the strategy with the highest probability of being cost-effective. In agreement with ICER results, MRI-only was extendedly dominated and had a low probability of being cost-effective across all CETs. Unconditional-CTM had zero probability of being cost-effective across all CETs.

**Figure 4:**
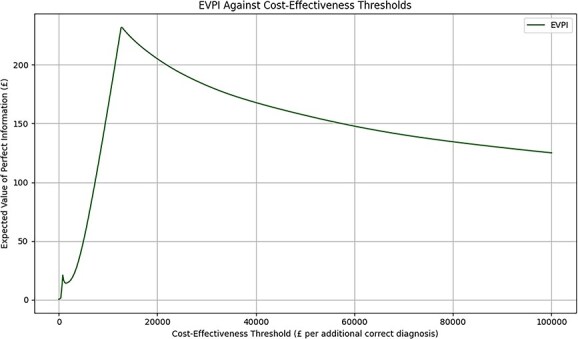
EVPI across a range of cost-effectiveness thresholds. Abbreviation: EVPI = expected values of perfect information.

The maximum EVPI of £231.92 per patient was noted at a CET threshold of £12 826 per correct diagnosis (Figure 4), with the cost of decision uncertainty being lower at all other CETs. This finding indicates that, from a purely economic perspective and at this CET, the maximum amount worth spending on further research to eliminate all uncertainty around the choice of diagnostic strategy is approximately £231.92 per patient.

## Discussion

The study compared the cost-effectiveness of 5 cross-sectional imaging strategies in the diagnosis of thoracolumbar IVDE in dogs in the UK. Of the 5 tested strategies and under study conditions, the efficient set of strategies included both conditional-CTM and conditional-MRI. At lower CETs, conditional-CTM was the most cost-effective strategy whereas conditional-MRI was cost-effective only at higher CETs. Magnetic resonance imaging (MRI)-only was extendedly dominated and rarely cost-effective. Computed tomography (CT)-only and unconditional-CTM were strictly dominated and never cost-effective under PSA, but CT-only may be cost-effective under low CET thresholds.

Cost–benefit analyses of diagnostic tests and treatments have been conducted extensively in human healthcare,^33–35^ but have been poorly investigated in veterinary medicine. In small animal practice, cost–benefit evaluations have been reported for procedures such as prophylactic fenestration^36^ and extracapsular stabilization for cranial cruciate ligament disease^37^ but no studies have investigated the cost-effectiveness of a veterinary diagnostic procedure. The findings of the study may be relevant during clinical decision-making, and aid in the choice of diagnostic imaging modality in the population of dogs suspected to have thoracolumbar IVDE. Although no cross-sectional imaging technique has been definitively proven superior, each is associated with distinct clinical and nonclinical advantages and disadvantages.^38^ The selective use of diagnostic imaging is important to improve patient care,^6^ and the study quantifies the economic impact of clinical decision-making in this patient population.

Magnetic resonance imaging has been referenced as the gold standard for diagnosis of IVDE in dogs, but this imaging modality is limited by prolonged acquisition times and added costs compared with CT-based techniques.^7^ The study showed that the incremental increase in sensitivity gained by an MRI-only strategy is not justified by its additional costs when compared with conditional imaging approaches using noncontrast CT as the initial imaging modality. Our results are in agreement with a previous study^39^ in that although MRI provides superior anatomical detail compared with other imaging modalities, its use leads to an escalation in costs with no demonstrable improvement in patient-centered or system-level outcomes. In radiology in human medicine, MRI has been found to be cost-effective in some scenarios but not others, depending on the clinical indication and available alternatives.^40,41^ The presence of both conditional-CTM and conditional-MRI in the efficient set supports the use of noncontrast CT as a cost-effective first-line imaging strategy for diagnosing thoracolumbar IVDE, in agreement with a previous study.^32^ Unconditional-CTM was strictly dominated by both conditional strategies and is therefore unlikely to be cost-effective as a routine strategy. Although CT-only was strictly dominated in PSA and therefore not cost-effective, it briefly appeared as the preferred strategy at very low CETs of < £728. This observation reflects a low willingness to pay rather than a true clinical advantage. In practice, CT-only may only be considered when MRI is unavailable and myelography cannot be performed because of a lack of expertise, materials, or owner consent. However, even in these scenarios, CT-only provides lower diagnostic accuracy and is generally suboptimal compared with conditional imaging strategies.

Both conditional-CTM and conditional-MRI were shown to be cost-effective, with each strategy demonstrating varying probabilities of being cost-effective under different CETs. Conditional approaches to diagnostic imaging allow efficient use of resources, reserving more invasive or expensive imaging modalities (CT-myelography or MRI) for patients with inconclusive initial results on first-line imaging. This approach optimizes the use of finite resources to deliver veterinary health care, especially in settings where cost is a key determinant of diagnostic access. The use of myelography and the associated adverse effects have been criticized previously.^10–12^ The study demonstrates that the additional costs of myelography and treatment of potential adverse events were not significant determinants of cost-effectiveness, and that CT-myelography remained cost-effective within a conditional imaging strategy. Nonetheless, the use of conditional-CTM as a strategy is contingent on the availability of trained personnel to perform myelography, which may have regional variability. The study did not consider any downstream clinical effects after adverse events that may be seen with myelography. Conditional imaging strategies have been investigated in human medical radiology, and have been shown to decrease the number of follow-up imaging studies with no loss of diagnostic accuracy.^42^ Cost-effectiveness thresholds, representing the maximum additional amount a key stakeholder is willing to pay for an incremental improvement in diagnostic accuracy, have not been formally defined in veterinary clinical decision-making. The empirical CET, of either the animal owner or a third-party insurance provider, remains unknown. As the first study to investigate the cost-effectiveness of diagnostic imaging in veterinary medicine, a wide range of CETs was explored. However, not all of these thresholds are likely to reflect real-world values. In human health care, universal CETs have yet to be defined^43,44^ although previous studies have suggested CETs for diagnostic testing to be between US $1000 and $2000,^45,46^ with other studies reporting CETs upward of £20 000.^19^ Inferentially, the realistic CET of a stakeholder in the veterinary setting is likely to be within, or below, these defined values, given the privately funded veterinary healthcare model. This conclusion, therefore, would suggest that conditional-CTM is likely the most cost-effective imaging strategy under real-world conditions. The ICERs in the study indicate how much extra it costs to obtain one additional correct diagnosis when choosing a more advanced imaging strategy over a less costly alternative. When these results are considered alongside the CEAC, they show which strategy offers the best value for money at different CETs. The EVPI estimates the value of eliminating remaining uncertainty in the study’s results, and determines whether further research to refine the results is financially worthwhile. These findings may be more relevant for future modeling or analytic studies instead of having direct clinical relevance.

The study conducted PSA within a decision-analytic framework using a simulated cohort of patients and model input parameters, which is widely accepted in cost-effectiveness modeling, particularly when direct comparative trial data are lacking.^19,41,47^ Nonetheless, the lack of clinical data and reliance on secondary data sources and probabilistic modeling meant that potential adverse patient effects of radiation,^48^ client preferences, institutional resource constraints, or broader system-level effects may not have been fully accounted for in the study. The study assumed that MRI had a diagnostic sensitivity of 100.0% instead of the reported minimum of 98.5%,^13^ and may have slightly overestimated the effectiveness of MRI-inclusive strategies. The study also assumed the use of high-field MRI, which is associated with different costs and image quality compared with low-field MRI.^49^ The study did not investigate all cross-sectional imaging modalities. The use of CT-angiography was unaccounted for in any of the investigated imaging strategies, because it has not been reported to add value to the diagnostic evaluation of acute myelopathy in dogs.^9^ The model input parameters in the study were estimated from a range of plausible values through PSA. Although primary data may be preferred, primary cost data also can be an imperfect source of information.^50^ Hence, PSA may better and more realistically reflect a plausible range of costs and diagnostic sensitivities encountered in clinical practice and in different patient populations. The cost parameters used in the study were estimated from a single country, and the results of this study may not be applicable in other countries where the cost differential between CT and MRI may be different. The model considered cost-effectiveness only from a diagnostic perspective, focusing on cost per correct diagnosis of IVDE. The robustness of the ICER estimate for the MRI-only strategy is limited by the low inclusion frequency of this strategy on the efficient frontier. Therefore, this estimate is likely to be unstable and should be interpreted with caution. The study assumes a clinical scenario in which thoracolumbar IVDE is the primary suspected diagnosis, which reflects the populations used in published diagnostic accuracy studies and does not consider other competing acute myelopathies. Accordingly, the cost-effectiveness results presented here are most applicable to this specific clinical context and may not be generalizable to diagnostic scenarios in which the pretest probability of IVDE is not high and when alternative thoracolumbar myelopathies are considered to be reasonably possible. Downstream treatment decisions and patient outcomes were outside the scope of the study. The use of MRI may offer prognostic information,^51,52^ but the prognostic utility of MRI has not been proven beyond reasonable doubt.^53,54^ From a treatment planning perspective, the use of MRI has been associated with planning of a larger hemilaminectomy window,^55^ but whether this approach leads to an improvement in patient outcomes is unknown. Despite these limitations, the use of PSA and explicit modeling of uncertainty provides a platform for evaluating competing imaging strategies. As the first application of cost-effectiveness analysis in veterinary diagnostic imaging, this model offers a foundation for future research and decision-making in clinical practice. Additional prospective studies to refine the cost–benefit of these imaging strategies and investigate downstream effects of imaging, their associated cost-effectiveness, and the impact on patient outcomes are necessary. Importantly, the study reported here does not suggest that MRI lacks value in the diagnostic evaluation of dogs with thoracolumbar myelopathy. However, within the narrow scope of the study, an MRI-only strategy was rarely cost effective.

In conclusion, conditional-CTM and conditional-MRI were the most cost-effective cross-sectional imaging strategies to diagnose thoracolumbar IVDE, among dogs where it was the most likely diagnosis. Conditional-CTM was more cost-effective than conditional-MRI over a lower range of CETs. An MRI-only strategy was rarely cost-effective. Noncontrast CT was the first-line imaging modality forming the basis of both dominant imaging strategies. In this decision-analytic modeling study, strategic use of cross-sectional imaging in the diagnosis of thoracolumbar IVDE was identified as having the potential to optimize the use of finite resources.

## Supplementary Material

aalag016_Supplemental_Files

## Off-label antimicrobial declaration

The author declares no off-label use of antimicrobials.

## Institutional animal care and use committee or other approval declaration

The author declares no institutional animal care and use committee or other approval was needed.

## Abbreviations

IVDE: intervertebral disc extrusion
CT: computed tomography
MRI: magnetic resonance imaging
PSA: probabilistic sensitivity analysis
ICER: incremental cost-effectiveness ratio
NMB: net monetary benefit
CET: cost-effectiveness threshold
CEAC: cost-effectiveness acceptability curve
EVPI: expected value of perfect information
UK: United Kingdom
