# Supplementary figures and images for: Comparative cost-effectiveness of cross-sectional imaging strategies in the diagnosis of intervertebral disc extrusion in dogs: a United Kingdom-based decision-analytic study
Source: J Vet Intern Med. 2026 Feb 23;40(1):aalag016. doi: 10.1093/jvimsj/aalag016 (PMC12927879; doi:10.1093/jvimsj/aalag016)

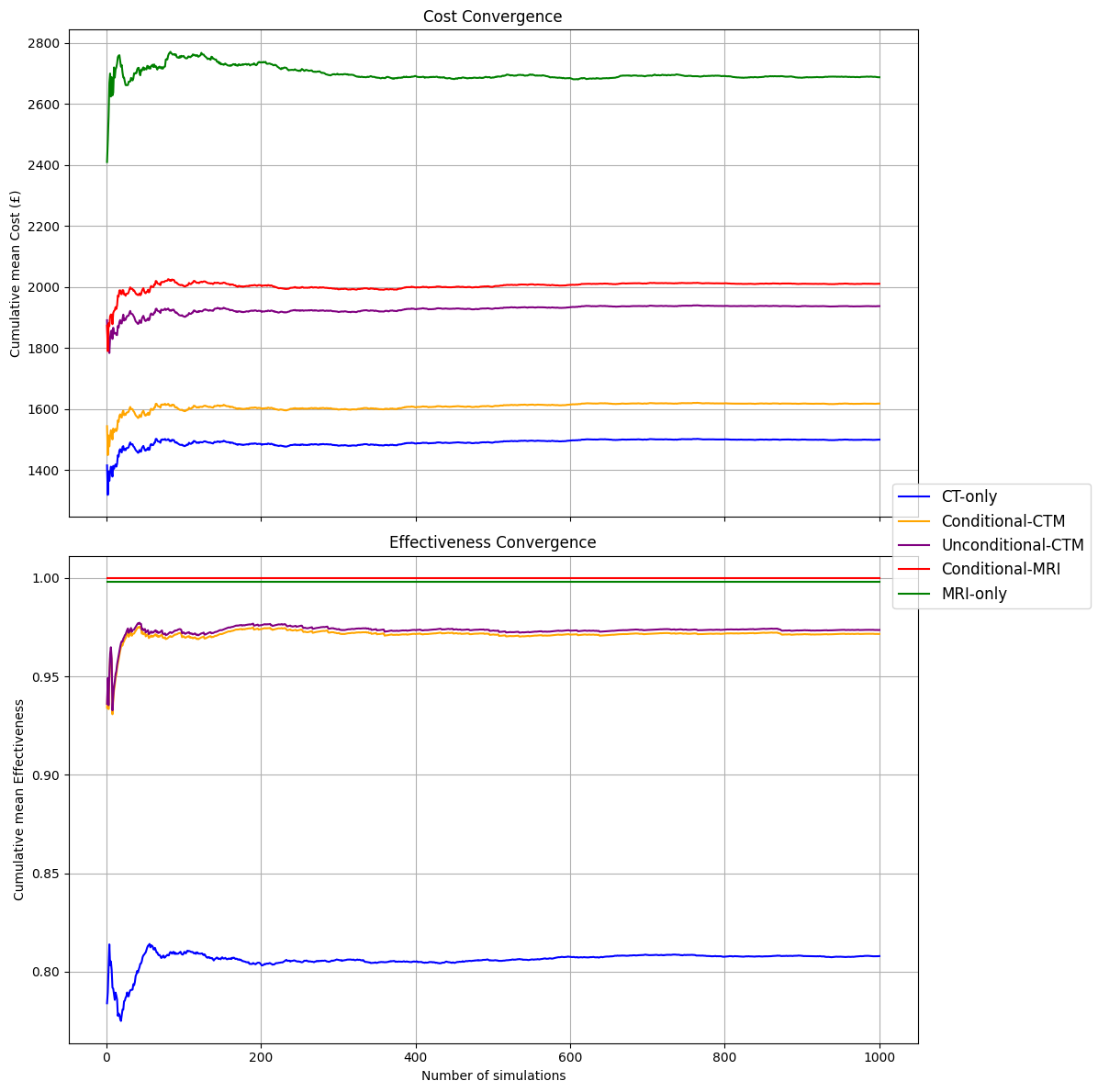

Supplement: aalag016_Supplemental_Files [file aalag016_supplemental_files.zip › supp_1_aalag016.png]

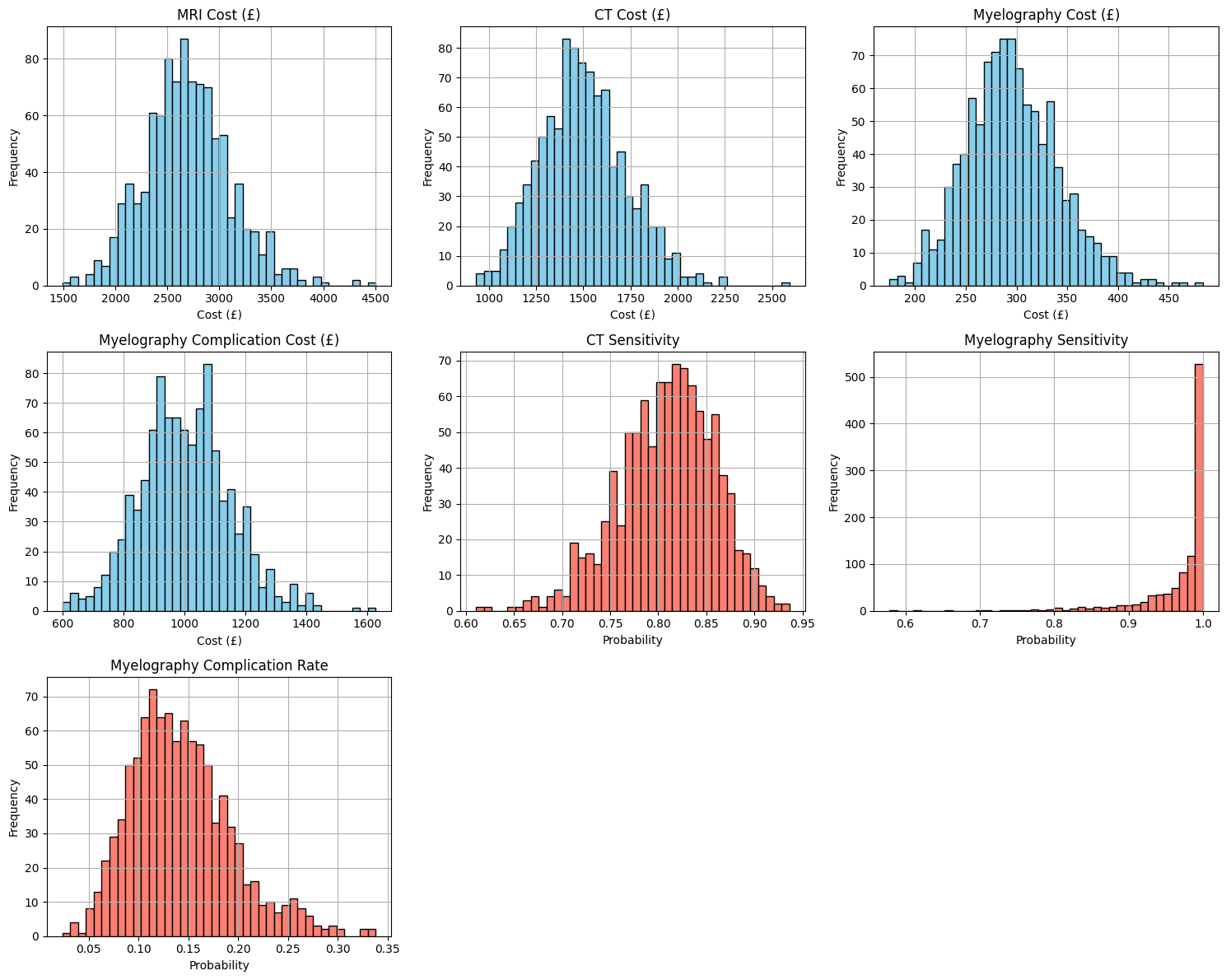

Supplement: aalag016_Supplemental_Files [file aalag016_supplemental_files.zip › supp_2_aalag016.png]
